# BCL-2 protein family: attractive targets for cancer therapy

**DOI:** 10.1007/s10495-022-01780-7

**Published:** 2022-11-07

**Authors:** Deeksha Kaloni, Sarah T Diepstraten, Andreas Strasser, Gemma L Kelly

**Affiliations:** 1 Blood Cells and Blood Cancer Division, Walter and Eliza Hall Institute of Medical Research, Melbourne, VIC Australia; 2 Department of Medical Biology, University of Melbourne, Melbourne, VIC Australia

**Keywords:** Apoptosis, BCL-2 protein family, Pro-survival BCL-2 proteins, Pro-apoptotic BCL-2 family members, BH3-only proteins, BH3-mimetic drugs

## Abstract

Acquired resistance to cell death is a hallmark of cancer. The BCL-2 protein family members play important roles in controlling apoptotic cell death. Abnormal over-expression of pro-survival BCL-2 family members or abnormal reduction of pro-apoptotic BCL-2 family proteins, both resulting in the inhibition of apoptosis, are frequently detected in diverse malignancies. The critical role of the pro-survival and pro-apoptotic BCL-2 family proteins in the regulation of apoptosis makes them attractive targets for the development of agents for the treatment of cancer. This review describes the roles of the various pro-survival and pro-apoptotic members of the BCL-2 protein family in normal development and organismal function and how defects in the control of apoptosis promote the development and therapy resistance of cancer. Finally, we discuss the development of inhibitors of pro-survival BCL-2 proteins, termed BH3-mimetic drugs, as novel agents for cancer therapy.

## Introduction

**Fig. 1:**
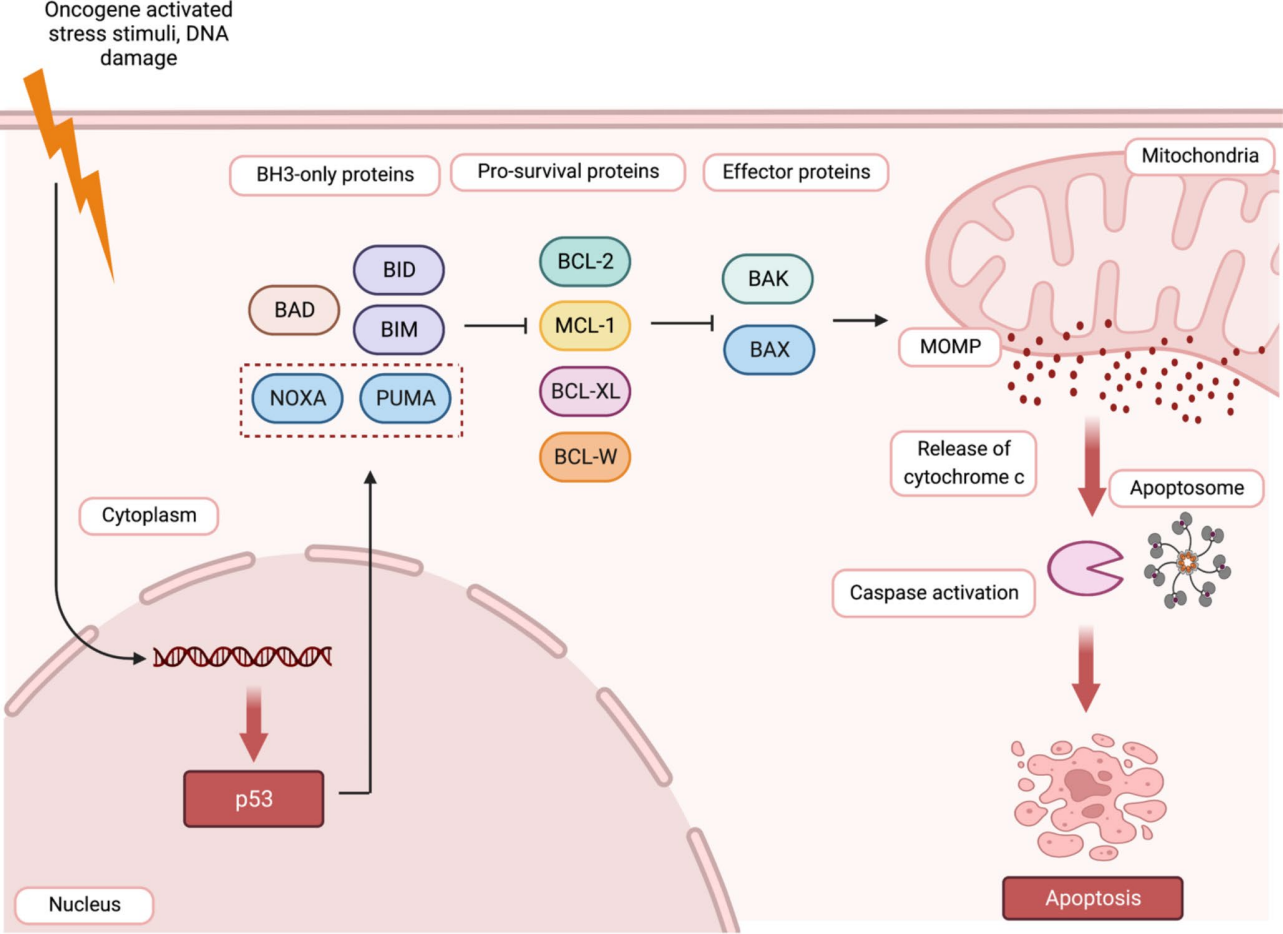
The intrinsic pathway of apoptotic cell death is controlled by the BCL-2 protein family. This pathway is activated in response to various stress stimuli, such as oncogene activation or DNA damage. This causes an increase in the levels of the BH3-only proteins (e.g., PUMA, NOXA, BIM, BID, BAD) through diverse transcriptional as well as post-transcriptional processes. For example, the genes for PUMA and NOXA are directly transcriptionally activated by the tumour suppressor TP53/TRP53 (indicated in the dashed red box). The BH3-only proteins bind to the pro-survival BCL-2 proteins (e.g., BCL-2, BCL-XL, MCL-1) with high affinity. This unleashes the pro-apoptotic effector proteins BAK and BAX from their restraint by the pro-survival BCL-2 family members. The effectors of apoptosis, BAX and BAK, are also reported to be activated directly by certain BH3-only proteins, such as PUMA, BIM, and t-BID (the caspase activated form of BID). The activation of BAX and BAK allows these proteins to oligomerise and form pores in the outer mitochondrial membrane. This results in outer mitochondrial membrane permeabilisation (MOMP) causing release of cytochrome c from the space between the inner and the outer mitochondrial membranes into the cytoplasm. Upon release into the cytosol, cytochrome c drives the formation of a heptameric complex of the apoptotic protease activating factor 1 (APAF-1), called the apoptosome, which triggers the caspase cascade that causes the ordered demolition of the cells undergoing apoptosis

Apoptosis is an important cellular phenomenon critical for the development, survival, and functioning of multi-cellular organisms [1]. Consequently, deregulation of apoptosis is commonly associated with a broad range of diseases, ranging from cancer to degenerative disorders [2]. There are two well-defined pathways to apoptosis - the mitochondrial, also known as the intrinsic, stress-induced or BCL-2 regulated pathway, and the death receptor-induced, also known as the extrinsic pathway [3]. Several proteins participate in the process of programmed cell death, including caspases (cysteinyl aspartate-specific proteases), their adaptors/activators and the BCL-2 (B-cell lymphoma/leukemia-2 gene) protein family members which constitute the critical regulators of apoptosis. The BCL-2 protein family members can be classified into three sub-groups based on their functions and amino acid sequence similarity. This includes the pro-apoptotic BH3-only proteins (BIM, BID, PUMA, BMF, NOXA, BIK, BAD, HRK), the pro-survival proteins (BCL-2, BCL-XL, BCL-W, MCL-1, A1/BFL-1) and the effectors of apoptosis (BAX, BAK, BOK) [4–6]. The interaction between the members of the BCL-2 protein subgroups determines whether a cell will undergo apoptosis or survive. In healthy cells the pro-survival BCL-2 proteins restrain the effectors of apoptosis, BAX and BAK, to safeguard their survival. In response to a broad range of stresses, such as nutrient or growth factor deprivation, oxidative stress, γ-irradiation and treatment with diverse cytotoxic drugs, the levels of the pro-apoptotic BH3-only proteins are increased through diverse transcriptional and/or post-transcriptional processes [6–8]. The BH3-only proteins can bind with high affinity to the pro-survival BCL-2 proteins, and this unleashes the effectors of apoptosis, BAX and BAK, from their restraint. Upon such activation, BAX and BAK oligomerise and form pores in the outer mitochondrial membrane, thereby causing mitochondrial outer membrane permeabilisation (MOMP) resulting in the release of apoptogenic factors from inside the mitochondria, including cytochrome *c* and SMAC/DIABLO [9–11](Fig. 1). Some BH3-only proteins, including PUMA, BIM and the activated form of BID, called tBID, have also been reported to be able to bind and thereby directly activate BAX and BAK [12], but whether this process is critical for apoptosis initiation has been challenged [13]. MOMP unleashes the cascade of caspases, that cleave hundreds of cellular proteins and thereby drive the ordered demolition of the dying cells [14]. The expression of the various BCL-2 protein family members is stringently controlled at the transcriptional, post-transcriptional, and post-translational levels [6, 15, 16].

The impact of abnormal over-expression of pro-survival BCL-2 proteins as well as abnormally reduced expression of pro-apoptotic BCL-2 family members on tumour development and the resistance of malignant cells to anti-cancer agents are well established [17–19]. Therefore, BCL-2 family members and their regulators are attractive targets for the development of anti-cancer therapeutics [20, 21]. This review describes the roles of the different BCL-2 family members in the normal development and functioning of multi-cellular organisms and the impact of their dysregulation in cancer. We also discuss therapeutic strategies to target these regulators of apoptosis for cancer therapy, for example using BH3-mimetic drugs which inhibit selective pro-survival BCL-2 proteins.

## The role of pro-survival BCL-2 proteins in organismal development and function

The different pro-survival members of the BCL-2 protein family exert distinct critical roles during organismal development and function. The differences between them are due in part to differences in their expression patterns [22]. BCL-2 is expressed in a broad range of haematopoietic cell subsets, melanocyte progenitors, certain epithelial cell populations in the embryonic kidney and in certain neuronal cell populations. The absence of BCL-2 in mice causes fatal polycystic kidney disease within ~ 30 days post-birth, premature greying of the coat and an abnormal reduction in mature B and T lymphocytes [23, 24]. These defects can all be prevented by the concomitant absence of the pro-apoptotic BH3-only protein BIM [25]. The relatively high levels of BCL-2 during early neurulation in mice (E4.5–8) suggest its role in preventing apoptosis at that stage. BCL-2 expression wanes after the neural tube is formed in the central nervous system (CNS), whereas high levels are maintained in the peripheral nervous system [26]. Notably, however, BCL-2-deficient mice do not have marked defects in the CNS [23, 24], indicating that its role in the survival of these cells can be effectively backed up by other pro-survival BCL-2 family members.

BCL-XL is expressed broadly during embryonic development and its levels are particularly high throughout neuronal ontogeny including in differentiating cells [27]. The absence of BCL-XL in mice leads to embryonic death around E13.5 as a consequence of defects in the survival of certain neuronal cell populations and erythroid progenitors [28]. The loss of BCL-XL causes aberrant apoptosis in post-mitotic immature neurons of the developing brain, spinal cord, and dorsal root ganglion, demonstrating its essential role in the survival of these cell populations [28]. Conditional gene deletion studies have shown that in adult mice BCL-XL is critical for erythropoiesis and the survival of certain cell populations in the kidney [29].

BCL-W is expressed in several tissues, such as the testes, colon, brain and certain myeloid and lymphoid cell populations [30]. BCL-W is essential for spermatogenesis. BCL-W deficient male mice display progressive testicular degeneration with apoptosis of Sertoli cells occurring soon after weaning [31, 32]. Furthermore, abnormal death of Leydig cells is seen in BCL-W deficient males starting at 3 months of age. This causes disruption of the architecture of the testes and sterility. BCL-W knockout mice are otherwise normal in all other cell types examined [32].

MCL-1 is expressed in a broad range of cell types both during embryogenesis and in mice after birth [33]. MCL-1 has a critical role during early embryonic development. Genetic studies revealed the dependency of several cell types on MCL-1 for survival [22]. *Mcl-1* gene knock-out mice die prior to implantation around embryonic day 3.5 [34]. Conditional gene knockout studies have shown that MCL-1 plays an essential role in hepatocytes, cardiomyocytes, neuronal cells, intestinal epithelial cells, mammary epithelial cells and several haematopoietic cell subsets [35–39]. Specifically, MCL-1 is required for the survival of haematopoietic stem and progenitor cells (HSPCs), the development of B as well as T cells and NK cells, the formation and maintenance of germinal-centre B cells, the development and survival of plasma cells (PCs) and immature erythroid cells [40–46].

A1/ BFL-1 is mainly expressed in haematopoietic cells, such as mitogen activated B and T cells, but its absence has only minimal impact on mice [47, 48]. *A1*-knockdown studies using in vivo expression of shRNAs in the haematopoietic system suggested a role for A1 in mast cell maturation [49], mature B cell survival [50] and early T cell development [51], but this was not replicated in studies of complete *A1* gene knockout mice [47].

## Regulation of the different members of the BCL-2 protein family

The balance between the various pro-survival and pro-apoptotic members of the BCL-2 protein family is crucial for normal embryonic development and tissue homeostasis after birth. The levels and activity of BCL-2 family members can be controlled through a broad range of transcriptional (transcriptional induction vs. repression), post-transcriptional (mRNA stability, effects of micro-RNAs or lncRNAs) and post-translational (e.g. phosphorylation, proteolytic processing and subcellular localisation) processes [6, 52].

The expression of pro-survival members of the BCL-2 protein family can be transcriptionally regulated by several transcription factors, such as E2F-1, the nuclear factor kappa B (NF-kB) family, and Janus kinase (JAK)-signal transducers and activators of transcription (STAT). Many of these transcription factors are activated by signalling from receptors for diverse cytokines (e.g. IL-2, IL-3, IL-4, IL -6, IL-7) [53] or the stimulation of antigen receptors on B as well as T cells [54].

The expression of BCL-2 protein can be transcriptionally regulated by the NF-κB transcription factor family [55] and by STATs [56]. The expression of BCL-2 is negatively regulated by the miRNAs, miR-15a, and miR-16-1, whereas the RNA binding protein nucleolin has been shown to increase BCL-2 expression by binding to the 3′-UTR, thereby enhancing *BCL-2* mRNA stability [57]. BCL-2 protein is a long-lived protein with a half-life of about 20 h [46], and it was reported that this can be impacted by phosphorylation at residue Ser-70 [58].

Like the BCL-2 protein, BCL-XL is also relatively stable with a half-life of ~ 20 h [59]. The expression of BCL-XL can be increased in response to a variety of stimuli, such as IL-2, IL-3, IL-6, granulocyte-macrophage colony-stimulating factor (GM-CSF), colony-stimulating factor-1 (CSF-l), leukaemia inhibitory factor (LIF), erythropoietin (EPO) as well as the stimulation of antigen receptors, which can all promote the survival and/or proliferation of several haematopoietic cell subsets [60]. The transcription factors ETS (erythroblastosis virus E26 oncogene homolog), REL/NF-KB, STAT and AP-1 have all been reported to transcriptionally upregulate expression of the gene encoding BCL-XL [60]. Activated RAS/mitogen-activated protein kinase (RAS/MAP kinase), integrin, vitronectin and hepatocyte growth factor signalling cascades have also been shown to cause an increase in the expression of BCL-XL [61]. The microRNAs miR-5-5p, miR-125b, miR140-5p, miR133a-3p, miR4300, miR-377 and hsa-let-7b-5p are all reported to modulate the expression of BCL-XL [62–64].

BCL-W can be transcriptionally regulated by several transcription factors, including NF-κB, MEF2 (myocyte enhancer factor 2), ETS-1 and ETS-2, and C/EBP (CCAAT/enhancer-binding protein) [65]. BCL-W expression is positively regulated by the TCF4 (β-catenin/transcription factor 4) complex and transgenic expression of either dominant-negative TCF4 (TCF4ΔN) or wild-type β-catenin resulted in downregulated or upregulated activity of the promoter for *BCL2L2* that encodes BCL-W, respectively [66]. Several miRNAs, including miR-29 and miR-122 [67], were shown to negatively regulate the expression of BCL-W by binding to the 3’-untranslated region (3’-UTR) of the *BCL-W* transcript[68]. A long non-coding RNA (lncRNA) RP11-436H11.5 functions as a competitive endogenous RNA, and was reported to sequester miR-335-5p which then causes an increase in the levels of BCL-W [68].

The expression of MCL-1 is regulated at the transcriptional, post-transcriptional and post-translational levels [69]. MCL-1 expression can be increased by many cytokines and growth factors, involving a range of signalling pathways [69]. Vascular endothelial growth factor (VEGF) and IL-6 regulate the expression of MCL-1 via autocrine signalling loops [70]. Activation of the MAPK/ERK (mitogen-activated protein kinase/extracellular signal-regulated kinase) signalling pathway reduces MCL-1 protein degradation and thereby increases its levels [71]. Activation of the NOTCH-1 signalling pathway induces the production of IL-6, thereby increasing the expression of MCL-1 [72]. MCL-1 protein levels are regulated by IL-15 and IL-22 through the JAK/STA3 [73] and phosphatidylinositol-3-kinase (PI3K) signalling pathways [74]. A broad range of miRNAs have been shown to downregulate the expression of MCL-1, including miR-26a [75], miR-15a, miR- 101 and miR-197 [76]. MCL-1 is a short-lived protein with a half-life of approximately 30 min [77]. Several E3 ubiquitin ligases, including MULE [78], SCFFbw7 [79], APC/C^Cdc20^ [80] and SCFB-TrCP [81], regulate the stability of the MCL-1 protein. These ubiquitin ligases prime MCL-1 for proteasomal degradation. Conversely, the de-ubiquitinases USP9X [82] and USP13 [83] have been reported to stabilise the expression of the MCL-1 protein. The PEST domain of MCL-1 contains many phosphorylation sites, including Thr-92, Thr-163, Ser-64, Ser-155, and Ser-159. Phosphorylation of residues in the PEST domain of MCL-1 (region rich in amino acids Proline (P), glutamic acid (E), serine (S) and threonine (T)) by protein kinases, such as CDK1/2 (cyclin- dependent kinase 1/2), GSK3 (glycogen synthase kinase-3), JNK (c-Jun N-terminal kinase) and ERK, has been reported to impact access to different E3 ligases and thereby affect the ubiquitination and stability of MCL-1 [84, 85].

The gene encoding BFL-1/A1 is a direct target of NF-kB transcription factors [86]. The PI3K and JAK/STAT signalling pathways have also been reported to regulate the expression of BFL-1/A1. BFL-1/A1 has a short half-life of ~ 15 min and this is at least in part due to its ubiquitination followed by proteasomal degradation [87, 88].

The expression of the pro-apoptotic BCL-2 family proteins is also highly regulated at the transcriptional, post-transcriptional and post-translational levels [16]. For example, the genes that encode the BH3-only proteins PUMA [89, 90] and NOXA [91] are directly transcriptionally upregulated by the tumour suppressor TP53, and consequently their expression is increased in response to cytotoxic stimuli that cause DNA damage and thereby activate TP53 [89, 91]. PUMA is also important in the response of cells to certain TP53-independent apoptotic stimuli, such as treatment with glucocorticoids or phorbol ester [92]. Of note, it is not well understood how these agents control PUMA expression. The *PUMA* gene contains binding sites for several transcription factors in its promoter region, exon 1 and intron 1. These transcription factors include TP53, as mentioned, its close relatives TP63 and TP73 which bind to the same response element [93, 94] and also c-MYC, FOXO3a (Forkhead box O3a) which can be activated by growth factor deprivation, C/EBP homologous protein CHOP, and E2F1, the latter two activated by ER stress [95, 96]. *Puma* transcription can be down-regulated as part of a negative feedback process during TP53 activation. In response to DNA damage, TP53 induces the expression of the transcriptional repressor SLUG, which inhibits TP53-mediated transcription of *Puma* [97]. Post-translational modifications can also regulate the expression of PUMA. For example, it has been reported that the levels of PUMA can be reduced through phosphorylation at certain sites, including Ser10, which promotes its proteasomal degradation [98].

The gene that encodes NOXA, *PMAIP1*, can be transcriptionally regulated by various transcription factors, including by the tumour suppressor TP53 [91] and also by HIF-1α, E2F-1, MYC, TP63 and TP73 [99]. HIF-1α causes an increase in the levels of NOXA protein under hypoxic conditions, thereby mediating cell death in a TP53-independent manner [100]. *NOXA* can also be transcriptionally induced in response to post-translational modifications of IRF-1, IRF-3 and CREB [101]. The NOXA protein can be degraded by the 26S proteasome after priming by K11-linked poly-ubiquitination [102] or through a ubiquitin-independent pathway [103].

Transcription of the *BCL2L11* gene, encoding BIM, has been reported to be regulated by FOXO3a [104], c-MYC [105], NF-Y[106], SMAD1/3[107], RUNX1-3[108], c-Jun [109] and RELA [110] although the importance of BIM regulation by the FOXO transcription factors has been questioned [111]. It has also been reported that the promoter for the gene encoding BIM can be epigenetically regulated through methylation of CpG dinucleotides [112]. BIM expression is also regulated by several miRNAs, such as the miR-106b~25 and miR-106a~363 clusters, and most prominently by the mir-17-92 cluster [113–116]. The activity and stability of the BIM protein is reported to be controlled by the phosphorylation of several residues. Phosphorylation can occur through a JNK-dependent mechanism, which promotes BIM dissociation from dynein light chain 1 (DLC1) [16, 117–119] and allows it to move to the mitochondria and induce BAK/BAX activation and apoptosis, or by the MAPK/ERK pathway that promotes BIM degradation and thereby increases cell survival [110]. The importance of the latter process has been questioned and there is evidence that ERK inhibits BIM mediated apoptosis not via a post-translational process but through direct transcriptional repression or induction of miRNAs that target the gene for BIM [120].

BMF expression can be transcriptionally regulated through the MAP kinase and AKT signalling pathways, for example in apoptosis that occurs during mammary epithelial morphogenesis [121]. The expression of BMF can also be epigenetically modulated at the promoter for its gene via CpG islands. Accordingly, treatment with histone deacetylase (HDAC) inhibitors causes a marked increase in the levels of BMF [122].

## Binding patterns of the BCL-2 family proteins

Apoptosis signalling is controlled by complex interactions between the pro-survival BCL-2 family members, the pro-apoptotic BH3-only proteins and the effectors of apoptosis, BAX and BAK. The pro-survival BCL-2 proteins can either directly inhibit BAX and BAK by binding to them or by binding to the BH3-only proteins, thereby preventing them from activating the effectors of apoptosis. BIM, PUMA and tBID (the caspase activated form of BID) can bind to all pro-survival BCL-2 proteins with very high affinity [123, 124]. BAD selectively binds to BCL-2, BCL-XL and BCL-W, whereas NOXA selectively binds to MCL-1 and A1 [123, 124]. BAX and BAK also differ in their ability to bind to the pro-survival BCL-2 proteins. BAX can interact with all pro-survival BCL-2 proteins, whereas BAK associates only with MCL-1 and BCL-XL [125]. In contrast, the effector protein BOK is not regulated by the pro-survival BCL-2 proteins or the BH3-only proteins; instead the activity of BOK appears to be regulated mostly by its levels of synthesis and degradation [126, 127].

## The role of BCL-2-family proteins in tumorigenesis

Evasion of apoptosis is one of the hallmarks of cancer [128]. Defects in the control of apoptosis that contribute to the development, expansion and therapy resistance of cancer can be caused by abnormally increased expression of pro-survival proteins or abnormally decreased expression of pro-apoptotic proteins.

### Aberrantly increased levels of pro-survival BCL-2 proteins

Abnormally high expression of pro-survival BCL-2 proteins is correlated with the development and poor prognosis of various cancers [4, 129, 130]. BCL-2, MCL-1, and BCL-XL are frequently over-expressed in lymphomas and leukaemias [20, 21, 131, 132]. The genomic regions containing the genes encoding MCL-1 and BCL-XL are somatically amplified in ~ 15% of diverse tumour types [133]. The abnormal over-expression of pro-survival BCL-2 family proteins can also be caused by chromosomal translocations or increased gene transcription [134]. However, it is also known that cellular dependence on distinct pro-survival BCL-2 family proteins does not always correlate with expression patterns. Studies using conditional gene targeting or inducible CRISPR platforms revealed that different malignant cells rely on the expression of distinct pro-survival BCL-2 proteins for their sustained survival, such as MCL-1 for MYC-driven lymphomas [135], even though their genes are not over-expressed owing to somatic copy number amplification or chromosomal translocation. This may be because their normal cellular counterparts rely on these same pro-survival BCL-2 proteins for their survival or because stresses present in malignant cells have imposed these dependencies.

The t(14;18) chromosomal translocation causes deregulated over-expression of BCL-2 in human follicular lymphoma (FL) [136, 137]. High levels of BCL-2 were also detected in several other haematological malignancies, including chronic lymphocytic leukemia (CLL), diffuse large B cell lymphoma (DLBCL) and mantle cell lymphoma [138–140] and in certain solid tumours, including subsets of brain, breast and lung cancer [141, 142]. Over-expression of BCL-2 greatly accelerates c-MYC driven lymphoma development in mice [143]. Moreover, over-expression of BCL-2 (or other pro-survival BCL-2 proteins) renders both malignant as well as non-transformed cells markedly resistant to diverse anti-cancer agents that kill cells in either a TP53-dependent [144] or TP53-independent manner [145].

Approximately 3% of human cancers of diverse origin carry somatically acquired amplification of the region that harbours the gene for BCL-XL [133]. It has been reported that BCL-XL plays a critical role in the progression of glioma [146] and breast cancer [147]. Human multiple myeloma (MM) cells as well as melanoma cells express high levels of BCL-XL [148, 149] and, accordingly, some of these malignant cells can be killed by treatment with inhibitors of BCL-XL, either on their own or more potently in combination with inhibitors of oncogenic kinases [150–152]. High levels of BCL-XL have also been observed in certain lymphomas, such as B cell non-Hodgkin lymphomas, FL, and DLBCL as well as T cell non-Hodgkin lymphomas [153]. Notably, EBV-associated T/NK cell lymphoma cells are dependent on BCL-XL for continued growth and survival [154]. High levels of BCL-XL can be detected in many colorectal cancers and, accordingly, inhibition of BCL-XL impairs adenoma outgrowth in vivo and enhances the efficacy of chemotherapy in colorectal cancer [155].

In gastric cancer, high levels of BCL-W have been reported to promote the survival, migration, and invasion of malignant cells [156]. Furthermore, BCL-W was observed in colorectal adenocarcinomas, with relatively higher levels detected in advanced-stage cancers as compared to localised tumours with better prognosis [157]. It has also been reported that certain lymphoma cells rely on BCL-W for sustained survival [158], but another study was not able to reproduce this finding [159].

MCL-1 is expressed at relatively high levels in many haematological malignancies, including MM and acute myeloid leukaemia (AML), as well as in cancers of the breast, pancreas, prostate, lung, and ovary [160–164]. Approximately 12% of human cancers of diverse origin carry somatically acquired amplifications of the region that harbours the *MCL-1* gene [133]. Transgenic mice over-expressing MCL-1 in haematopoietic cells develop B lymphoid [165] or myeloid malignancy, albeit with low incidence and long latency [165, 166]. Moreover, MCL-1 over-expression greatly accelerates the development of c-MYC driven lymphoma [166]. Studies using inducible gene deletion revealed that a broad range of cancer cells, including AML [167], MYC driven B cell lymphomas [135], T cell lymphomas and lung cancer caused by loss of TP53 or mutations in Notch [168–170] require MCL-1 for sustained survival and growth. These findings indicate that MCL-1 could be an attractive target for cancer therapy [164] .

### Aberrantly decreased levels of pro-apoptotic BCL-2 family proteins are observed in diverse human cancers

The reduction of pro-apoptotic members of the BCL-2 family has also been implicated in the development and therapy resistance of cancer. The levels of the BH3-only proteins BIM and/or PUMA are abnormally low in several cancers [174, 175]. For example, ~ 40% of human Burkitt lymphomas express very low levels of the mRNAs for BIM and/or PUMA, and this was ascribed to epigenetic silencing of their genes [174, 176]. Reduced expression of PUMA has also been reported in subcutaneous melanoma, and this correlated with poor prognosis [177]. The downregulation of BIM due to deletion or hyper-methylation of the gene was reported in mantle cell lymphoma [178] and DLBCLs [176, 179]. Finally, abnormally low levels of NOXA and/or BIM were observed in colon cancer and small-cell lung cancer [180].

There are reports of loss of BAX expression in human cancers, including endometrial and colon cancers [181, 182]. However, the combined loss of BAX and BAK, which would be required to render cells resistant to apoptosis because of the extensive functional overlap of these effectors of apoptosis [127, 183], is only rarely seen in human cancer (e.g. some AML cells) [167], probably because four alleles would need to be mutated to achieve this.

## The deletion of pro-apoptotic members of the BCL-2 family can accelerate tumour development in mice

Several studies using gene-targeted mice demonstrated that the absence of pro-apoptotic BCL-2 family members promotes tumour development and renders malignant cells resistant to a broad range of anti-cancer agents. Mice lacking either PUMA [92, 184] or BIM [185], two of the BH3-only proteins that can inhibit all pro-survival BCL-2 proteins, do not spontaneously develop tumours, but mice lacking both of these critical initiators of apoptosis develop plasma cell-like tumours with advanced age [186]. In cells genetically engineered to express oncogenes, the impact of loss of pro-apoptotic BCL-2 family members is even more pronounced. The individual loss of the genes encoding PUMA or BIM (even loss of one allele) substantially accelerates MYC-driven pre-B/B cell lymphoma in mice carrying an *Eµ-MYC* transgene [187, 188]. The loss of PUMA was also shown to cooperate with the oncogenes *H-Ras* or *E1A* in the neoplastic transformation of fibroblasts in culture [189]. Finally, loss of BMF or NOXA has been shown to accelerate *γ*-irradiation induced thymic T cell lymphoma development in mice [122, 190].

Since the genes that encode the pro-apoptotic proteins PUMA and NOXA are both direct transcriptional targets of TRP53, it was hypothesised that mice lacking PUMA and NOXA (and therefore lacking the capability to undergo TRP53-mediated apoptosis) would develop tumours at the same rate as TRP53-deficient mice. However, in contrast to TRP53-deficient mice which all develop tumours prior to 300 days of age (in the absence of an engineered oncogenic driver), the PUMA-deficient as well as PUMA/NOXA double-deficient mice do not develop cancer spontaneously on a C57BL/6 genetic background [188, 191]. This demonstrates that loss of the pro-apoptotic function of TRP53 alone is not sufficient to cause tumour development, i.e. other cellular processes activated by TRP53, such as the coordination of DNA damage repair, may be even more critical for its ability to suppress tumorigenesis [192].

## Defects in the intrinsic apoptotic pathway render malignant cells resistant to a broad range of anti-cancer therapeutics

Resistance to anti-cancer therapy contributes to poor clinical outcomes. Defects in the intrinsic apoptotic pathway, owing to over-expression of pro-survival BCL-2 proteins or the abnormal reduction of pro-apoptotic BCL-2 family members, render both malignant as well as non-transformed cells profoundly resistant to a broad range of anti-cancer therapeutics. This was first demonstrated when lymphoid cells from *BCL-2* transgenic mice were found to be resistant to several DNA damage-inducing anti-cancer agents and glucocorticoids [144]. Accordingly, increased levels of BCL-2 expression have been correlated with resistance to several anti-cancer drugs, including 5-fluorouracil, adriamycin and mitomycin, in gastric cancer [193], cisplatin in ovarian cancer [194] and doxorubicin in osteosarcoma and chondrosarcoma [195, 196].

The over-expression of BCL-XL has also been reported to protect tumour cells from a broad range of chemotherapeutic drugs [197, 198]. High levels of BCL-XL driven by STAT5 have been implicated in the resistance of BCR/ABL^+^ chronic myelogenous leukaemia (CML) to apoptosis [199]. Moreover, in a cisplatin-resistant patient cohort of ovarian cancer, 61.5% of samples displayed over-expression of BCL-XL [200]. A study using a nude mouse tumour xenograft model showed that BCL-XL over-expression rendered ovarian cancer cells resistant to cisplatin, paclitaxel, topotecan and gemcitabine [200].

High levels of MCL-1 have also been associated with the resistance of a broad range of malignant cells to chemotherapeutic agents [164]. Overexpression of MCL-1 causes resistance to various conventional chemotherapeutic agents, such as cisplatin in ovarian cancer cells [201], lapatinib in a human colon cancer cell line [202], rituximab in B-cell malignancies [203] and prednisone in MLL-rearranged infant acute lymphoblastic leukemia [204]. Of note, the downregulation of MCL-1 levels restores the effectiveness of these anti-cancer drugs in several cancer cell lines [205, 206].

Experiments using mice lacking different BH3-only proteins identified which of these proteins are critical for cell killing by different anti-cancer agents. PUMA is required for the killing of cells by DNA damage inducing agents that activate TRP53 [92, 184]. Of note, the combined loss of PUMA, NOXA, and BIM renders MYC-driven lymphoma cells almost completely resistant to killing by DNA damage inducing anti-cancer agents, such as etoposide or cyclophosphamide [207]. BIM and PUMA both contribute to the killing of lymphoid cells by glucocorticoids [92, 185] and BIM is also needed for the killing of malignant cells by inhibitors of oncogenic kinases, such as the BCR-ABL inhibitor Gleevec in chronic myeloid leukaemia (CML) [150, 151, 208]. Malignant as well as non-transformed cells lacking both BAX and BAK are profoundly resistant to all anti-cancer agents tested [183], demonstrating that these effectors of apoptosis are essential for such cell killing and that the killing of cells by these drugs is mediated to a large extent by the induction of apoptosis.

## Therapeutic interventions that directly target the apoptotic machinery

**Fig. 2:**
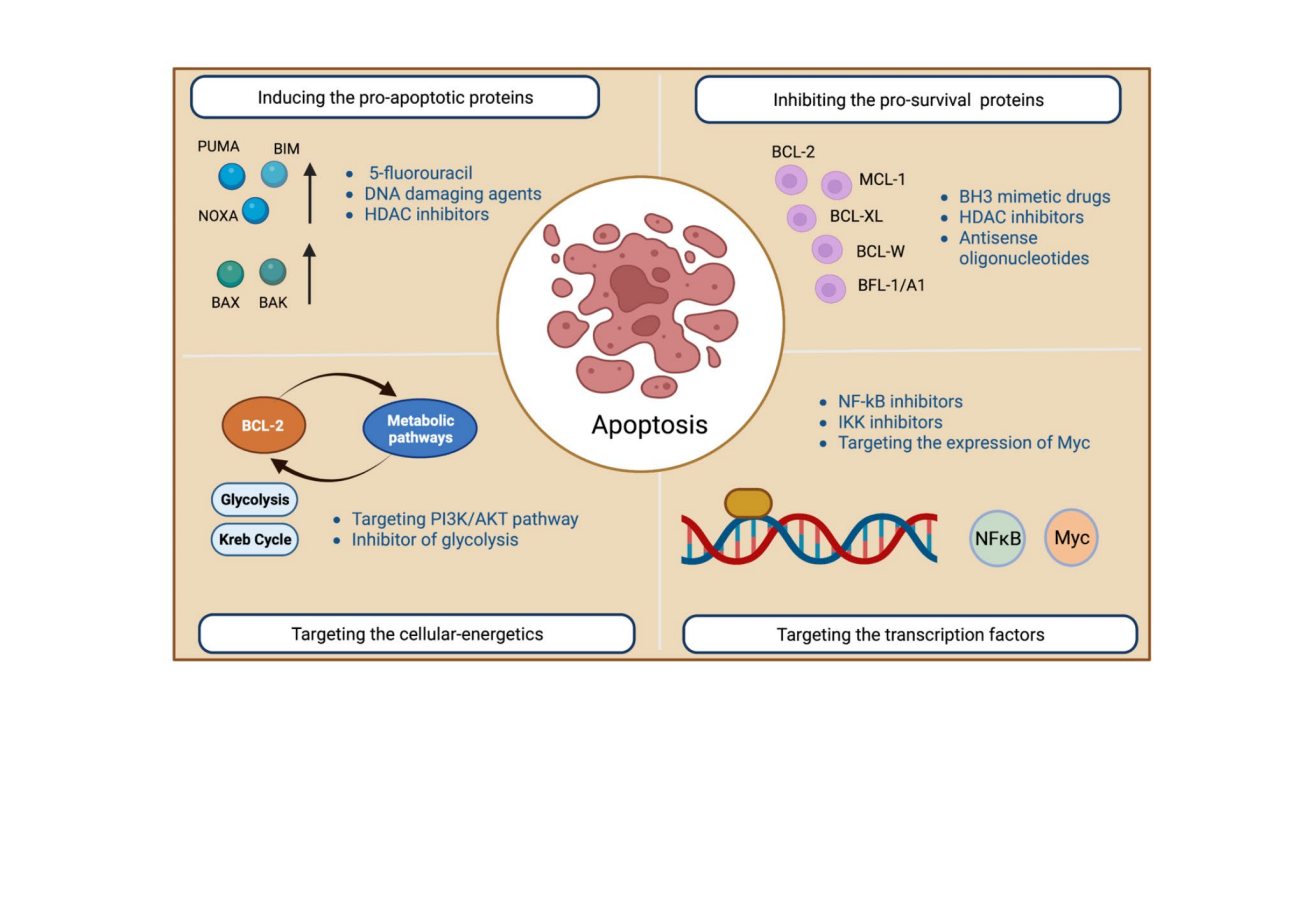
Potential strategies targeting the intrinsic apoptotic pathway either by directly targeting the pro-apoptotic or the pro-survival BCL-2 family members or by targeting metabolic pathways and signal transducers to induce apoptosis by causing an increase in pro-apoptotic BH3-only proteins and/or a decrease in the pro-survival BCL-2 family members

There are several different approaches to induce apoptosis of cancer cells for therapy (Fig. 2). The discovery that BH3-only proteins are critical for the initiation of apoptosis triggered by diverse anti-cancer agents, and that genetic deletion of distinct pro-survival BCL-2 proteins can effectively kill certain types of malignant cells, gave rise to the concept that pharmacological inhibitors of pro-survival BCL-2 proteins that mimic the function of BH3-only proteins could be effective in cancer therapy [20, 21]. This led to programs by pharma and biotechnology companies to generate small molecules that mimic the function of pro-apoptotic BH3-only proteins, known as the BH3-mimetic drugs [21]. Since non-transformed cells in healthy tissues also depend on pro-survival BCL-2 proteins for their survival (see above), a notable issue for the clinical use of BH3-mimetic drugs is their on-target toxicity [20, 21].

The first BH3-mimetic compounds ABT- 737 and its orally available derivative ABT-263 (navitoclax) inhibit BCL-2, BCL-XL, and BCL-W. They can induce apoptosis in a broad range of cancer-derived cell lines in vitro and delay the growth of certain tumours in vivo in tumour transplant models [152]. ABT-263/navitoclax was the first BH3-mimetic drug to be tested in patients [209]. ABT-263/navitoclax proved effective in CLL patients in clinical trials but dose-limiting thrombocytopenia, due to the dependence of platelets on BCL-XL for survival [210], hampered the progression of this agent in the clinic [211]. ABT-199 (venetoclax) was therefore developed as a BH3-mimetic drug that is a highly selective BCL-2 inhibitor that potently induces apoptosis in BCL-2 dependent malignant cells [212]. Venetoclax is highly effective in patients with relapsed or refractory CLL [52, 213]. Remarkably, the combination of venetoclax with rituximab (antibody against CD20) led to complete remissions in 51% of CLL patients, with disease-free survival persisting for up to 2 years after completion of therapy [214]. In high-risk relapsed/refractory AML patients, administration of venetoclax in phase 2 clinical trials resulted in complete response/complete response with incomplete blood recovery (CR/CRi) in 19% of patients [215]. Combination therapies for AML including venetoclax are proving even more effective. Considering the clinical benefits of venetoclax as a monotherapy, as well as in combination with standard-of-care anti-cancer drugs, it has now been approved by the FDA and several other regulatory authorities worldwide for the treatment of patients with CLL or AML [20, 21, 216, 217].

Many cancer cells have been shown to depend on BCL-XL for their sustained survival and proliferation, prompting the development of BCL-XL-specific BH3-mimetic drugs. WEHI-539 was the first compound to specifically target BCL-XL [218]. Additional structure-guided design led to the development of A-1155463 and A-1331852, which are both also selective for BCL-XL. A-1155463 exhibited anti-tumour activity in a xenograft model of small cell lung carcinoma (SCLC) in immune-deficient mice [219]. A-1331852 was shown to potently kill several cancer-derived cell lines on its own and cooperates with a broad range of anti-cancer agents in vitro [220]. However, at present, no BCL-XL specific inhibitors have been approved for clinical use, and clinical trials are progressing slowly because of the predicted on-target toxicity to platelets.

Abnormally increased expression of MCL-1 can drive tumorigenesis and often confers a poor prognosis. Therefore, several MCL-1 inhibitors have been developed and assessed in pre-clinical studies, these include S63845 [171], A-1210477 [221], AMG176 [172] and AZD5991 [173]. The in vitro and in vivo potential of the tool compound S63845 was explored in pre-clinical studies in haematological malignancies, such as MM, AML, CML, and c-MYC-driven Burkitt lymphoma [171]. S63845, either alone or in combination with inhibitors of oncogenic kinases, was found to be moderately effective in certain solid tumours, such as breast cancer and prostate cancer [171, 222] and SCLC derived cell lines that are express high levels of MCL-1 but low levels of BCL-XL [223]. There have been limited details on the potency of MIK665/S64315, the related compound that has entered clinical trials. The MCL-1 inhibitor AMG176 has been found to be effective in diverse haematological malignancies [172] and in certain solid tumour derived cell lines, such as breast cancer and non-small cell lung cancer [224]. AZD599, another MCL-1 specific BH3-mimetic drug, was also shown to be effective for the treatment of MM in mouse models, and its effect can be enhanced by co-treatment with the BCL-2 inhibitor venetoclax or the proteasome inhibitor bortezomib [173]. Until now, six MCL-1 inhibitors have entered clinical trials, but some of these trials have been halted due to on-target cardiac toxicity [225, 226]. This toxicity was predicted from genetic studies that had shown that MCL-1 is critical for the survival of cardiomyocytes [37, 227].

The survival of many cancer cells is safeguarded not by a single pro-survival BCL-2 protein but rather by two or even more of these proteins. Hence, effective killing of such cancer cells will require two or more BH3-mimetic drugs, or combined treatment with one BH3-mimetic drug plus one or several standard-of-care anti-cancer agents that cause an increase in BH3-only proteins which then inhibit the pro-survival BCL-2 proteins that are not targeted by the BH3-mimetic drug used [20]. The tolerability of such therapies will need to be carefully determined. For example, it is unlikely that inhibitors of MCL-1 and BCL-XL can be combined safely in patients, given that mice lacking only single alleles of the genes encoding MCL-1 and BCL-XL die on the day of birth because of severe craniofacial and several other defects [228]. Of note, the combination of an MCL-1 inhibitor with a BCL-2 inhibitor was shown to be tolerable in mice, providing a synergistic response that was able to overcome drug resistance in models of DLBCL [229]. AML derived cell lines were more potently killed by the combination of an MCL-1 inhibitor (S63845) and a BCL-2 inhibitor (venetoclax) than by treatment with either agent alone [229]. Other than the BH3-mimetic drugs, antisense oligonucleotides [230] and HDAC inhibitors that can lead to repression of expression of the BCL-2 pro-survival proteins [231] might be a promising approach in targeting the pro-survival members of the BCL-2 protein family.

Since the pro-apoptotic BH3-only proteins are the critical initiators of apoptosis, there should also be a focus on developing novel therapeutic strategies that increase the expression of these pro-apoptotic proteins. Such therapeutic interventions could be used alongside BH3-mimetic drugs to increase their effectiveness. This could be achieved by either using drugs that can boost expression of the BH3-only proteins directly or by developing drugs that can inhibit negative regulators of their expression. It is known that many conventional chemotherapeutic drugs do induce expression of the BH3-only proteins, particularly those drugs that can induce DNA damage. Colon cancer cells exposed to 5-fluorouracil-based anti-cancer therapy display elevated expression of PUMA and BIM and high induction of these proteins is correlated with a better prognosis of the patients [180]. Decreased levels of BIM have been correlated with poor response to diverse inhibitors of oncogenic kinases in several cancers [232] and therefore strategies to boost BIM expression would be anticipated to increase sensitivity both to inhibitors of oncogenic kinases and to BH3-mimetic drugs. Towards targeting negative regulators of BH3-only protein expression, efforts are underway to find TRP53 independent regulators of these proteins. Since the expression of PUMA and BIM can be suppressed by epigenetic modifications, drugs that target epigenetic regulators, such as HDAC inhibitors, might be beneficial for upregulating these initiators of apoptosis, thereby increasing the effectiveness of anti-cancer therapy [233, 234].

There is considerable evidence that cellular metabolism can impact the levels of certain pro-survival as well as pro-apoptotic BCL-2 family members. Of note, alterations in tumour cell metabolism were shown to potentiate the ability of malignant cells to evade apoptosis. The PI3K/AKT pathway provides a link between cell proliferation and cell metabolism. In cancer, the PI3K/AKT pathway is frequently aberrantly activated, for example due to the expression of oncogenic kinases, loss of PTEN as well as mutation or amplification of the gene for PI3K, promoting glucose metabolism [235–237]. The oncogenic kinase BCR-ABL regulates expression of the glucose transporter 1 (GLUT 1) via the PI3K/AKT pathway [235]. Thus, several inhibitors of oncogenic tyrosine kinases have been developed that act at least in part by inhibiting the PI3K/AKT pathway to suppresses glucose metabolism [238–240]. Moreover, directly targeting the glycolysis pathway with 2-deoxyglucose (2-DOG) was shown to enhance cisplatin induced killing of ovarian cancer cells [241]. Deprivation of nutrients, such as glucose or amino acids, causes a substantial decrease in MCL-1 levels because this causes a reduction in protein translation via activation of AMPK-activated protein kinase (AMPK), leading to the inhibition of mTOR [242]. Reduced glucose metabolism as a consequence of cytokine deprivation causes an increase in the levels of pro-apoptotic PUMA and BIM [243, 244] leading to BAX/BAK mediated apoptosis [245]. These approaches therefore tip the balance between pro-apoptotic and anti-apoptotic proteins towards the induction of apoptosis and the elimination of cancer cells.

NF-κB transcription factors are critical regulators of both the adaptive and innate immune systems [246]. They can promote cell survival by modulating the expression of certain pro-survival BCL-2 family members, most notably BCL-XL and A1/BFL-1 [54, 247]. These findings suggest that inhibitors of NF-kB signalling, such as inhibitors of IKK, an upstream activator of NF-kB, may be used to enhance BH3-mimetic drug or chemotherapeutic drug induced killing of cancer cells by reducing the levels of pro-survival BCL-2 proteins.

MYC is a helix-loop-helix-leucine zipper protein that, as a heterodimer with MAX, binds to a palindromic E-box element CACGTG in DNA and thereby upregulates the expression of specific target genes. In non-transformed cells, the expression of MYC relies on mitogenic signals and MYC is critical for cell volume growth and cell proliferation [248]. Approximately 70% of cancers express high levels of MYC, owing to chromosomal translocations (e.g., in Burkitt lymphoma), genomic amplifications or oncogenic signals. Studies using mice with a regulatable *Myc* transgene showed that MYC-driven tumour cells die when MYC is removed [249]. Deregulated MYC expression can also enhance the predisposition of cells to undergo apoptosis in response to stress, such as growth factor deprivation [250]. This involves transcriptional processes relating to the MYC relative MNT that causes an increase in BIM [251]. Accordingly, genetic loss of BIM or PUMA reduces MYC-driven apoptosis [187, 252]. Once the mechanisms by which MYC causes an increase in BIM and PUMA are understood, it may become possible to manipulate this process to increase the levels of these pro-apoptotic BH3-only proteins in malignant cells for therapeutic benefit, either alone or in combination with anti-cancer agents, such as BH3-mimetic drugs.

Since BAX and BAK are the critical effectors of apoptosis, it is possible that plasma membrane permeable agents that can activate these proteins could be effective in cancer therapy. However, the safety of such approaches would need to be considered carefully since most, if not all, non-transformed cells express either BAX and/or BAK and would therefore also be targets of such agents. Possibly, activators of BAX and BAK can only be administered safely to patients when conjugated to antibodies or ligands that will direct them preferentially to malignant cells. Such conjugate approaches are already being explored to increase the safety and utility of BH3-mimetic drugs that have considerable on-target toxicities to non-transformed cells, particularly those targeting MCL-1 or BCL-XL [20]. Finally, since the intrinsic and the death receptor activated apoptotic pathways are distinct, albeit converging on the activation of effector caspases [3], it is expected that BH3-mimetic drugs (activating the intrinsic apoptotic pathway) and activators of death receptors, such as the TRAIL receptors, would cooperate in killing malignant cells [253]. Again, the tolerability of such approaches will need to be tested rigorously.

## Concluding remarks

The BCL-2 protein family members constitute the crucial regulators of apoptosis. Abnormalities in the expression of pro-survival or pro-apoptotic members of the BCL-2 protein family can promote tumour development and render malignant cells resistant to anti-cancer therapy. The field has developed a detailed understanding of the control of apoptosis and how the different subgroups of the BCL-2 family proteins interact with each other. This understanding has enabled the development of novel anti-cancer drugs, called BH3-mimetics, that can directly activate the apoptosis machinery by inhibiting pro-survival BCL-2 proteins. These compounds have shown efficacy in pre-clinical studies, and some have entered clinical trials for cancer therapy, with the BCL-2 specific inhibitor venetoclax FDA approved for the treatment of patients with CLL or AML. Current efforts are aimed at developing effective and tolerable treatment schedules for the BH3-mimetic drugs that inhibit MCL-1 or BCL-XL and to discover which other anti-cancer agents can be combined with these drugs to achieve effective and safe cancer therapy. We believe that gaining a clearer understanding of how the expression of the pro-apoptotic BH3-only proteins is regulated may lead to insights that can be harnessed to develop novel therapeutics that enhance the expression of these initiators of cell killing. Such agents would be expected to cooperate with BH3-mimetic drugs and standard chemotherapeutics in killing malignant cells.

## Data Availability

As this is a review article, no new data are presented, and all published data are referenced.
